# Awareness of and Preferences for Preexposure Prophylaxis (PrEP) among MSM at High Risk of HIV Infection in Southern China: Findings from the T2T Study

**DOI:** 10.1155/2021/6682932

**Published:** 2021-04-02

**Authors:** Weiying Chen, Yi Ding, Jianghao Chen, Peipei Zhao, Zhenyu Wang, Xiaojun Meng, Tianjian Jia, Heping Zheng, Bin Yang, Zhenzhou Luo, Huachun Zou

**Affiliations:** ^1^Department of Dermatology and Venereology, Nanshan Center for Chronic Disease Control, Shenzhen 518054, China; ^2^Jane Addams College of Social Work, University of Illinois at Chicago, Chicago IL 60601, USA; ^3^Department of Medical Statistics, School of Public Health (Shenzhen), Sun Yat-sen University, Shenzhen 510080, China; ^4^Department of AIDS/STD Control and Prevention, Wuxi Center for Disease Control and Prevention, Wuxi 214000, China; ^5^Dermatology Hospital, Southern Medical University, Guangzhou 510080, China; ^6^Kirby Institute, University of New South Wales, Sydney 1466, Australia

## Abstract

This study is aimed at investigating the awareness of and preferences for oral and long-acting injectable HIV preexposure prophylaxis (PrEP) and their associated factors among men who have sex with men (MSM) at high risk of HIV infection in southern China. A cross-sectional survey was conducted among 603 MSM who were recruited through a cohort study called the T2T Study at three sexual health clinics in Guangzhou, Shenzhen, and Wuxi, China, from 2017 to 2018. We collected information on HIV-negative participants' awareness of and willingness to use PrEP and its potential correlations. Univariate and multivariate logistic regressions were used for data analyses. A total of 550 HIV-negative MSM were enrolled in the study. Less than half of at-risk MSM (43.1%) had heard of PrEP before, and the rate of overall willingness to use PrEP was 65.8%, while MSM were more willing to use daily oral PrEP than long-acting injectable- (LAI-) PrEP (62.2% vs. 38.5%). MSM who had university degrees or above (aOR = 1.55, 95% CI: 1.01-2.37), used condoms during last anal sex (1.52, 1.01-2.29), and tested 3 times or more for HIV (2.45, 1.10-5.47) were more likely to be aware of PrEP. MSM who had use of gay dating apps (1.51, 1.02-2.23), ever participated in HIV- or sexually transmitted disease (STD-) related studies (1.91, 1.24-2.94), and had heard of PrEP (3.06, 2.06-4.54) were more willing to use any regimen of PrEP. MSM at high risk of HIV infection had low awareness of PrEP and moderate willingness to use PrEP. Further studies of the implementation and promotion of PrEP targeting at-risk MSM should be performed.

## 1. Introduction

Men who have sex with men (MSM) are disproportionately affected by HIV. According to the Joint United Nations Programme on HIV/AIDS (UNAIDS), the number of new HIV infections globally in 2018 was approximately 1.7 million (1.4 million-2.3 million), where gay men and other MSM accounted for 17% [1]. In China, the overall prevalence of HIV among MSM was 8.0% in 2015 and has continued to increase [2], demonstrating the need for a more effective HIV prevention strategy. Preexposure prophylaxis (PrEP) is recommended by the World Health Organization (WHO) as an effective way to prevent HIV transmission among HIV-negative MSM [3, 4]. The most popular HIV PrEP is Truvada, a single tablet containing tenofovir disoproxil fumarate and emtricitabine that protects HIV-negative individuals from acquiring HIV. Daily oral PrEP showed promising results in decreasing HIV acquisition. A study among 2499 MSM and transgender women who have sex with men in six countries (Peru, Ecuador, South Africa, Brazil, Thailand, and the United States) found that HIV daily oral PrEP reduced incident HIV infection by 44% [5]. A recent study [6] reported that HIV incidence was 74% lower among 5,447 PrEP initiators in generalized HIV epidemic settings in Kenya and Uganda. Using modelled estimates, a study [7] found that 1,700-5,200 new HIV infections could be averted per year among high-risk young men and women if they were to take PrEP in Malawi, Mozambique, and Zambia. However, poor adherence to daily PrEP would impair the efficacy of daily oral PrEP and remains a concern among MSM [8, 9]. Long-acting injectable- (LAI-) PrEP that requires less frequent dosing is being studied as an alternative method to daily oral HIV PrEP [10–12]. In the US, a higher proportion of daily oral PrEP users prefer LAI-PrEP than the proportion of nonusers due to the inconvenience of daily oral PrEP [13]. A cross-sectional study in China reported that 76% versus 54% of MSM were willing to use LAI-PrEP and daily oral PrEP, respectively [14].

Truvada (emtricitabine 200 mg/tenofovir disoproxil fumarate 300 mg, FTC/TDF) was recently approved in China [15]. Considering that PrEP will soon be available and China is forming its guidelines on PrEP use, it is imperative to understand the acceptability and feasibility of both daily oral PrEP and LAI-PrEP, especially among MSM at high risk of HIV infection. According to the guidelines of the countries where PrEP is approved, PrEP is for individuals without HIV who are at risk of acquisition from sex or injection drug use, including MSM [16]. The risk of HIV infection was one of the important criteria for defining PrEP candidacy among MSM. Previous studies [17] also showed that MSM who have at least one STI in their lifetime were more likely to be aware of PrEP, and MSM with high sexual risk behaviours were more willing to accept PrEP [18–20]. However, existing PrEP studies in China have mainly focused on general HIV-negative MSM; however, those at high risk of HIV infection should be prioritized [21]. PrEP-related research focused on awareness, acceptability, and preferred regimens among MSM at high risk of HIV where infection is scarce. The objective of this study was to investigate the awareness of and willingness to take PrEP and preferred regimens among MSM at high risk of HIV infection in southern China.

## 2. Materials and Methods

### 2.1. Participants and Procedures

Data in this manuscript were extracted from the baseline data of the T2T Study, which was a randomized controlled trial that evaluated the impact of automated text message reminders on HIV or sexually transmitted disease (STD) testing behaviours among MSM in China. The study protocol was published previously [22]. The baseline survey was conducted from January to August 2017. Participants from three sexual health clinics in three cities in southern China, namely, Guangzhou, Shenzhen, and Wuxi, were enrolled. At enrolment, all male clients who visited the sexual health clinics were given a flyer with study information, including that it is a study for MSM. The clients who were interested in the study were introduced to the research assistant. After a brief introduction of the study, participants completed a computer-assisted self-interview to screen for eligibility and provide consent. Although MSM as a group are at much higher risk for HIV transmission than the general population, individual MSM are at various levels of risk for HIV transmission. According to a large sample of 47,231 MSM recruited from 61 cities across China [23], just over 40% had 1 or 0 male sex partners in the past 6 months. HIV prevalence was 3.7% vs. 5.8% in MSM with ≤1 vs. ≥2 male sex partners in the past 6 months. According to the WHO [3] and US CDC PrEP guidelines [16], PrEP should target those at considerable risk for HIV infection, not everyone in the key population. Therefore, we only recruited MSM who were at high HIV risk. To be eligible for this study, participants needed to meet the following criteria: (1) be 18 years old or above and have (2) had at least two male anal sex partners, or unprotected anal sex with nonregular partners, or had been diagnosed with STDs, including gonorrhoea, syphilis, anogenital warts, genital herpes, and chlamydia trachomatis, in the past 6 months. Blood, urine, and anal swabs were collected to test for HIV, syphilis, gonorrhoea, chlamydia trachomatis, and anogenital warts. Upon completion of the investigation, participants received an electronic mobile phone credit of CNY50 (USD 7) for their costs of time, travel, knowledge, and biological samples. MSM who tested HIV-positive were excluded from this analysis.

### 2.2. Measurement

#### 2.2.1. Background Characteristics

The survey collected sociodemographic information on age, education, monthly income, and history of STDs. A history of STDs was based on a self-reported STD diagnosis in the past 6 months, including gonorrhoea, syphilis, anogenital warts, genital herpes, and chlamydia trachomatis. Alcohol consumption in the past 6 months was classified by the frequency of drinking, assessed with two binary variables. Participants were also asked to report their drug use. Drugs in this study included ecstasy, methamphetamine, heroin, hemp, RUSH, zero capsule, Viagra, and other drugs. Using any one of these drugs in the past 6 months was classified as “Yes.” Participants were also asked about the HIV status of current regular or casual sex partners.

#### 2.2.2. Sexual Behaviours

In our study, sexual behaviours refer to anal sex among MSM unless otherwise specified. Participants were asked about the number of regular and casual sex partners in the past six months, condom use during last anal sex, sex with women, and the use of mobile phone dating apps targeting gay individuals. Such dating apps include Blued, Jack'd, and other popular social media platforms among Chinese MSM. Participants who had ever used dating apps to seek sexual partners in the past 6 months were classified as “Yes.”

#### 2.2.3. Testing of HIV, STDs, and Other Related Issues

Participants' testing behaviours included the testing frequency of HIV and STDs in the past 12 months. Participants were also asked about the history of circumcision and the experience of HIV- or STD-related studies. Participants who had ever participated in research about PrEP, innovative testing strategies, condoms, or other new prevention tools and approaches were treated as MSM with the experience of HIV- or STD-related studies.

#### 2.2.4. PrEP-Related Variables

To investigate awareness and overall willingness to use PrEP, a brief explanation of PrEP was provided before the following questions. “HIV preexposure prophylaxis (PrEP) is designed for people who have not yet been infected with HIV but are at high risk of HIV infection. It requires users to take the pill every day. The HIV incidence among users who adhere to daily PrEP can be reduced by more than 80%. Scientists are currently developing long-acting injectable- (LAI-) PrEP. Injection once every 1-2 months will potentially give protection similar to daily oral PrEP. However, LAI-PrEP will cause severe pain at the injection site. Daily oral PrEP has been used for several years and is priced at approximately CNY 6,000 (USD 888) per month. LAI-PrEP is still under research, and its market price is not yet available.” Two items assessed awareness and overall willingness to use PrEP: “Have you ever heard of PrEP before?” (Yes, No) and “Will you choose to use PrEP in the future?” (Yes, No). To investigate the willingness to use daily oral PrEP and LAI-PrEP, one single item was used to measure the preferred regimens of PrEP. Participants were asked, “Daily oral PrEP or LAI-PrEP, which do you prefer?” Responses were “only daily oral PrEP,” “only LAI-PrEP,” and “no preference.” Participants who answered “only daily oral PrEP” or “no preference” were classified into the willing to use daily oral PrEP group, and those who answered “only LAI-PrEP” and “no preference” were classified into the willing to use LAI-PrEP group. To investigate the reasons for willingness and unwillingness to use PrEP, the participants who chose to use PrEP were further asked, “Why do you choose to use PrEP?” with the responses including at high risk of HIV infection, partners at high risk of HIV infection, to reduce risk, and other reasons. The participants who refused to use PrEP were asked, “Why do you refuse to use PrEP?” with responses including being too expensive, being at low risk of HIV infection, concerns about drug resistance, trypanophobia, concerns about side effects, poor medication adherence, and other reasons. Participants could simultaneously select multiple options in the two questions above. To investigate the frequency of PrEP that participants could adhere to if they were to use PrEP, the adaptable frequency of using daily oral PrEP was measured by the question, “If you choose to use daily oral PrEP, how often can you take it?” The response options were daily, 2-5 days a week, and 1 day a week. Adaptable frequency of daily oral PrEP was categorized as nondaily when they responded 2-5 days a week and 1 day a week. The adaptable frequency of using daily oral PrEP was measured by the question, “If you choose to use LAI-PrEP, how often can you receive an injection?” and the response options were once a month, once every 2 months, and once every 3 or more months.

### 2.3. Data Analysis

Descriptive statistics were used to analyse sociodemographics, sexual behaviours, and HIV/STD testing. PrEP outcomes, including awareness of PrEP, overall willingness to use PrEP, willingness to use daily oral PrEP, and willingness to use LAI-PrEP, were treated as dependent variables. Univariate logistic regression was used to identify associations between the PrEP outcomes and the categorical variables. All factors with *P* < 0.10 in univariate logistic regression or considered clinically relevant were then included in the multivariable logistic regression models, adjusting for potential confounders. The adjusted odds ratios (aORs) and their 95% confidence intervals (95% CIs) were calculated. All analyses were conducted in IBM SPSS Statistics (version 21, SPSS Inc., Chicago, IL, USA), and two-tailed *P* < 0.05 was considered statistically significant.

## 3. Results

### 3.1. Background Characteristics

Of 603 participants enrolled, 53 MSM were excluded for testing HIV positive. As a result, 550 MSM were included in this analysis. The median age was 26 (IQR 23-31) years. The majority of participants had a university degree or above (67.3%, 370/550), earned >CNY 5,000 (USD 740) per month (59.5%, 327/550), and were single (62.4%, 343/550). Less than ten percent (8.4%, 46/550) of the participants reported a history of STDs. Over two-thirds (70.7%, 389/550) reported alcohol consumption habits, and more than one-fifth (25.6%, 141/550) used drugs in the past six months. Over three-quarters (75.1%, 413/550) of the participants were uncertain about the HIV status of their current sex partners (Table 1).

### 3.2. Sexual Behaviour Characteristics

In the past 6 months, over half of the participants had more than one regular sexual partner (52.7%, 290/550), and most of them had more than one causal sexual partner (90.7%, 499/550). Nearly three-quarters of MSM used a condom during the last anal sex (74.4%, 409/550). Approximately 34.2% of the participants reported sex behaviours with women. The majority of MSM (62.7%, 345/550) used gay dating apps to seek sex partners (Table 1).

### 3.3. Testing of HIV and STDs and Other Related Issues

Over one-fifth (23.3%, 128/550) of the participants had no HIV testing in the past 12 months, while over half (54.9%, 302/550) of the participants had no STD testing in the past 12 months. Approximately 16.7% (92/550) of the participants ever had a circumcision. Approximately thirty percent (28.7%, 158/550) of the participants had ever joined HIV- or STD-related studies (Table 1).

### 3.4. PrEP-Related Variables

Less than half of the participants (43.1%, 237/550) had heard of PrEP before this study. After being informed of PrEP, the overall willingness to use PrEP was 65.8% (362/550), while 62.2% (342/550) of the participants showed a willingness to use daily oral PrEP, 38.5% (212/550) showed a willingness to use LAI-PrEP, and 34.9% (192/550) accepted both daily oral PrEP and LAI-PrEP. Among 362 participants who showed willingness to use PrEP, 41.4% (150/362) preferred oral PrEP only, 5.5% (20/362) preferred LAI-PrEP only, and the majority of them (307/362, 84.8%) aimed to reduce the risk of HIV infection to a minimum. Among 188 participants who were unwilling to use PrEP, concerns about expensive prices (79/188, 41.0%) and side effects (82/188, 43.1%) were the main reasons hindering the willingness to use PrEP. Over half (52.9%, 181/342) of the participants who chose daily oral PrEP preferred nondaily frequency of using daily oral PrEP, while approximately half (50.5%, 107/212) of the participants who chose LAI-PrEP preferred once every 2 months and once every 3 or more months frequency of using LAI-PrEP (Table 2).

### 3.5. Factors Associated with Awareness of PrEP

In the univariate analysis, education, sex with women, condom used during last anal sex, use of gay dating apps in the past 6 months, and HIV testing in the past 12 months were significantly associated with awareness of PrEP (all crude odds ratios are shown in Table 3). In the multivariable model, MSM who had university degrees or above (*aOR* = 1.55, 95% CI: 1.01-2.37), used condoms during last anal sex (*aOR* = 1.52, 95% CI: 1.01-2.29), and tested 3 times or more for HIV in the past 12 months (*aOR* = 2.45, 95% CI: 1.10-5.47) were more likely to have prior awareness of PrEP (Table 3).

### 3.6. Factors Associated with the Willingness to Use PrEP

In the univariate analysis, alcohol consumption was only associated with the willingness to use LAI-PrEP. Using gay dating apps in the past 6 months was associated with the overall willingness to use PrEP and the willingness to use daily oral PrEP. Ever participating in HIV- or STD-related studies had heard that PrEP was associated with all three dependent variables (the overall willingness to use PrEP and the willingness to use daily oral PrEP and LAI-PrEP; all crude odds ratios are shown in Table 4). In the multivariable model, MSM who had used gay dating apps in the past 6 months (*aOR* = 1.39, 95% CI: 1.02-1.97), ever participated in HIV- or STD-related studies (*aOR* = 1.90, 95% CI: 1.24-2.92), and had heard of PrEP (*aOR* = 2.96, 95% CI: 2.06-4.54) were more likely to show willingness to use any regimen of PrEP. MSM who had ever participated in HIV- or STD-related studies (*aOR* = 1.59, 95% CI: 1.06-2.39) and had heard of PrEP (*aOR* = 2.75, 95% CI: 1.90-4.00) were more likely to show willingness to use daily oral PrEP. MSM who had ever participated in HIV- or STD-related studies (*aOR* = 1.51, 95% CI: 1.03-2.21) and had heard of PrEP (*aOR* = 2.00, 95% CI: 1.41-2.85) were more likely to show a willingness to use LAI-PrEP (Tables 4, 5).

## 4. Discussion

Our study investigated awareness of and willingness to use PrEP and preferred regimens among MSM at high risk of HIV infection in China, contributing to the literature on PrEP in developing countries. Overall, the results demonstrated that MSM at high risk of HIV infection lacked awareness of PrEP (43.1%) and had moderate willingness to take PrEP (65.8%). We also found that more MSM preferred daily oral PrEP than LAI-PrEP. The main reason for MSM to use PrEP was to minimize the risk of HIV infection, while the main barriers were high cost and concerns about side effects.

The level of awareness of PrEP in our study was close to that in a study that recruited MSM from a gay-friendly health consulting service centre (52.7%, 276/524) [24], which was much higher than that in other studies that recruited MSM online, with a PrEP awareness rate ranging from 7.4% to 22.4% [25–27]. One possible explanation is that health care centres are an important source of intervention information for MSM [28]. MSM who seek health services in clinics or health centres may have more opportunities to learn about PrEP. However, compared with studies in Australia (77%, 954/1251) [29], where PrEP was available and recommended, or Brazil (61.3%, 728/1270) [20], the awareness rate in our study was lower. One of the most straightforward ways to increase awareness of PrEP is to expand the media coverage of PrEP among MSM [20]. It should be noted that there are still challenges for routine PrEP implementation in China, such as traditional cultural beliefs on medicine and HIV stigma, barring the increase in PrEP awareness, and uptake among MSM at high risk of HIV infection [30]. Similar to some other studies [29, 31], MSM with higher education levels may have a higher HIV prevention awareness, and they would be more likely to take the initiative to learn about PrEP. MSM who tested for HIV more frequently had higher awareness of PrEP [31]. The association between HIV testing behaviours and PrEP awareness may be related to a higher risk perception among MSM who tested frequently. Our results suggested that if there is a promotion of PrEP for MSM at high risk of HIV in China, it may be necessary to engage with HIV-negative men who have lower education, no consistent condom use, and less HIV testing behaviours, as those MSM were less likely to know about PrEP.

In our study, the willingness to use PrEP among at-risk MSM was at a moderate to high level, and it was higher than a study that recruited at-risk MSM for actual uptake of PrEP in the US (60.5%, 337/557) [32], but it was close to some studies that recruited MSM at normal risk of HIV infection for perceived uptake of PrEP in Beijing [26] and western China [33] (willingness rates were 67.8% and 64.0%, respectively). Although the virtual acceptance of PrEP in China ranges from 7.4% to 84.9% [14, 26, 27], one study conducted in Shanghai reported that the rate of actual uptake of PrEP was only 2.5% [34]. This result suggested that the perceived willingness to use PrEP among MSM of normal risk and high risk was higher than the actual rate of willingness to take PrEP in China. In fact, low and slow uptake of PrEP is common around the world [35, 36] due to a lack of knowledge and availability of PrEP. The gap between actual and perceived uptake of PrEP must be narrowed in the future. Moreover, some researchers have begun to modify the guidelines [37] or find a tool to identify optimal PrEP candidates [38]. Identifying and prioritizing high-risk MSM may facilitate an increase in the willingness of MSM to use PrEP and other intensive HIV prevention interventions.

Of note, the utilization of mobile phone technologies is a potential way to disseminate PrEP-related information [39]. Similar to one study conducted in Brazil, MSM who used apps for sexual encounters were more willing to use PrEP [40], indicating that MSM who had HIV risk behaviours were more likely to show a willingness to use PrEP. However, we found no association between participating in HIV- or STD-related studies and awareness of PrEP, while both factors were related to willingness to use PrEP. One explanation is that participating in HIV or STD research was not the main resource for PrEP knowledge, but those groups of MSM had a higher interest in PrEP once it was recommended to them.

MSM in our study showed higher interest in daily oral PrEP (62.2%, 342/550) than LAI-PrEP (38.5%, 212/550), which is consistent with some previous studies [41, 42]. However, in some other studies, LAI-PrEP was more attractive [14, 43]. Both options have some advantages and disadvantages. Daily PrEP already exists and may be less demanding, but adherence may be difficult. LAI-PrEP is more convenient and easier to adhere to but painful when injected [44]. The diverse results suggested that the choice of PrEP regimens depended on different population characteristics and settings [45]. Regardless of the forms of PrEP, acceptability and users' ability to adhere to it have a significant impact on the success of PrEP [46]. PrEP should only be prescribed to those patients who can adhere to it [47]. However, only 41% of users persisted on PrEP across the entire 2-year period of research [48]. In our study, we found that almost half of at-risk MSM had little confidence in their ability to adhere to daily oral PrEP, but they seemed to be more confident in their ability to adhere to LAI-PrEP. This finding reflected the advantages and shortcomings of the two forms of PrEP.

In our study, the majority of at-risk MSM who showed a willingness to use PrEP expected to reduce HIV infection risk to a minimum and MSM who had refused to use PrEP were mainly worried about its side effects and unaffordable price. The side effects of PrEP are mild to moderate nausea, vomiting, and diarrhoea, and its adverse effects were limited according to current clinical trials of PrEP [49]. Learning from other studies, a lower cost of PrEP raises the willingness to use PrEP, and it is expected that the cost of PrEP will drop in the future [25]. Our results suggested that formulating publicity strategies towards eligible at-risk MSM should focus on the effectiveness, safety, and cost of PrEP.

Our study had several limitations. First, MSM were not asked about the specific knowledge of and the ways they know about PrEP. Awareness does not necessarily mean understanding. Investigations about ways to learn about PrEP would have allowed us to find a feasible way to design a highly effective promotion strategy that is conducive to the implementation of PrEP. Future studies should add more detailed questions to assess the knowledge and ways of learning about PrEP among at-risk MSM. Second, we only assessed the virtual uptake of PrEP among at-risk MSM attending sexual health clinics. Perceived willingness does not always translate into actual uptake because the implementation of PrEP faces a series of challenges. For example, the Chinese people believe that all medicines are poisonous and tend to refrain from taking any medicine when they do not have symptoms. More studies concentrating on behaviours should be performed following clinical studies. Third, questions about preferred regimens of PrEP were not detailed enough, and we did not collect the awareness of daily oral PrEP and LAI-PrEP separately. Fourth, mentioning that injectable PrEP causes severe pain at the injection site may have potentially led to a bias in the decision on PrEP preferences among participants. However, the trial HPTN 077 [50] found LAI-PrEP (cabotegravir) was well tolerated with mild to moderate pain and that severe injection reactions were common but infrequently led to product discontinuation. However, our study has successfully shown the awareness of and willingness to use PrEP among MSM at high risk of HIV infection in China. These results shed light on the findings of other studies and could be a reference for future studies about PrEP in China.

## 5. Conclusions

MSM at high risk of HIV infection have a low awareness of PrEP and moderate to high willingness to use PrEP. PrEP requires more promotion focusing on at-risk MSM who have lower education, do not practice consistent condom use, and have less HIV testing behaviours. MSM at high risk of HIV infection showed higher interest in daily PrEP. Implementation studies on the effectiveness of PrEP targeting MSM at high risk in China are needed.

## Figures and Tables

**Table 1:** Sociodemographic characteristics and sexual behaviours of MSM in southern China (*N* = 550).

Variables	Response categories	Frequency	Percentage (%)
Age (years)	18-24	233	42.4
	25-30	162	29.4
	>30	155	28.2
Education	Middle school or below	180	32.7
	University or above	370	67.3
Monthly income (CNY)	<2,000	60	10.9
	2,000-4,999	163	29.6
	5,000-9,999	231	42.0
	>9,999	96	17.5
History of STDs	No	504	91.6
	Yes	46	8.4
Alcohol consumption	No	161	29.3
	Yes	389	70.7
Drug use	No	409	74.4
	Yes	141	25.6
Self-reported HIV status of current sex partners^a^	Uncertain	413	75.1
	HIV negative	108	19.6
	HIV positive	21	3.8
Number of regular sex partners in the past 6 months	0	260	47.2
	1-2	211	38.4
	≥3	79	14.4
Number of casual sex partners in the past 6 months	0	51	9.3
	1-2	253	46.0
	≥3	246	44.7
Condom use during last anal sex	No	141	25.6
	Yes	409	74.4
Sex with women	No	362	65.8
	Yes	188	34.2
Ever used gay dating app in the past 6 months	No	205	37.3
	Yes	345	62.7
HIV testing in the past 12 months	No	128	23.3
	1-2 times	290	52.7
	3 times or more	132	24.0
STD testing in the past 12 months	No	302	54.9
	1-2 times	188	34.2
	3 times or more	60	10.9
Circumcision	No	458	83.3
	Yes	92	16.7
Ever participated in HIV- or STD-related studies	No	392	71.3
	Yes	158	28.7

CNY: Chinese Yuan, CNY 2,000 equal to USD 296 and CNY 9,999 equal to USD 1,512; STDs: sexually transmitted diseases; CDC: Centers for Disease Control. ^a^The last sex partners who are in regular or causal relationship with participants; 8 participants refused to disclose their partners' HIV status leading to missing values.

**Table 2:** Awareness, willingness, and preferences for oral and long-acting injectable HIV preexposure prophylaxis (PrEP) among MSM in southern China (*N* = 550).

Variables	Response categories	Frequency	Percentage (%)
Heard of PrEP	No	313	56.9
	Yes	237	43.1
Willingness to use PrEP	No	188	34.2
	Yes	362	65.8
Willingness to use daily oral PrEP	No	208	37.8
	Yes	342	62.2
Willingness to use LAI-PrEP	No	338	61.5
	Yes	212	38.5
Reasons for willingness to use PrEP^a^	At high risk of HIV infection	66	18.2
	Partner/s at high risk of HIV infection	36	9.9
	To reduce risk	307	84.8
	Other reasons^b^	16	4.4
Reasons for unwillingness to use PrEP^a^	Too expensive	79	41.0
	At low risk of HIV infection	43	22.3
	Concerns about drug resistance	32	16.5
	Trypanophobia	14	7.4
	Concerns about side-effects	82	43.1
	Poor medication adherence	45	23.4
	Other reasons^c^	37	19.7
Adaptable frequency of using oral PrEP^d^	Non-daily	161	47.1
	Daily	181	52.9
Adaptable frequency of using LAI-PrEP^d^	Once every 3 or more months	75	35.4
	Once every 2 months	32	15.1
	Once a month	105	49.5

LAI: long-acting injectable; PrEP: preexposure prophylaxis. ^a^Multiple-choice question. The number of participants who were willing to use PrEP was 362, and the opposite was 188. ^b^Other reasons included curiosity and recommendation from friends. ^c^Other reasons included job, family, and ease of suspicion. ^d^The number of participants who were willing to use oral PrEP was 342, and the number of participants who were willing to use LAI-PrEP was 212.

**Table 3:** Factors associated with awareness of PrEP among MSM in southern China (N =550).

Variables	Response categories	Awareness of PrEP	
		Crude OR (95% CI)	Adjusted OR (95% CI)
Age (years)	18-24	1.0	
	25-30	0.80 (0.53, 1.20)	
	>30	0.95 (0.63, 1.43)	
Education	Middle school or below	1.0	1.0
	University or above	1.90 (1.31, 2.76)^∗∗^	1.55 (1.01, 2.37)^∗^
Monthly income (CNY)	<2,000	1.0	
	2,000-4,999	0.68 (0.38, 1.23)	
	5,000-9,999	0.67 (0.38, 1.19)	
	>9,999	1.00 (0.53, 1.91)	
History of STDs	No	1.0	
	Yes	1.02 (0.55, 1.87)	
Alcohol consumption	No	1.0	
	Yes	1.09 (0.75, 1.58)	
Drug use	No	1.0	
	Yes	1.09 (0.74, 1.60)	
Self-reported HIV status of current sex partners^a^	Uncertain	1.0	
	HIV negative	0.87 (0.57, 1.34)	
	HIV positive	0.78 (0.32, 1.93)	
Number of regular sex partners in the past 6 months	0	1.0	
	1-2	1.15 (0.79, 1.65)	
	≥3	1.35 (0.81, 2.23)	
Number of casual sex partners in the past 6 months	0	1.0	
	1-2	0.64 (0.35, 1.17)	
	≥3	0.78 (0.43, 1.42)	
Condom use during last anal sex	No	1.0	1.0
	Yes	1.66 (1.12, 2.48)^∗^	1.52 (1.01, 2.29)^∗^
Sex with women	No	1.0	1.0
	Yes	0.60 (0.42, 0.87)^∗∗^	0.78 (0.52, 1.17)
Ever used gay dating app in the past 6 months	No	1.0	1.0
	Yes	1.53 (1.08, 2.19)^∗^	1.29 (0.89, 1.87)
HIV testing in the past 12 months	0	1.0	1.0
	1-2 times	1.43 (0.93, 2.20)	1.71 (0.80, 3.65)
	3 times or more	2.09 (1.27, 3.45)^∗∗^	2.45 (1.10, 5.47)^∗^
STD testing in the past 12 months	0	1.0	
	1-2 times	0.87 (0.60, 1.26)	
	3 times or more	0.96 (0.55, 1.68)	
Circumcision	No	1.0	
	Yes	0.78 (0.49, 1.23)	
Ever participated in HIV- or STD-related studies	No	1.0	
	Yes	1.24 (0.85, 1.79)	

CI: confidence interval; CNY: Chinese Yuan, CNY 2,000 equal to USD 302 and CNY 9,999 equal to USD 1,512; STDs: sexually transmitted diseases; CDC: Centers for Disease Control; PrEP: preexposure prophylaxis. ^a^The last sex partners who are in a regular or causal relationship with participants; 8 MSM refused to disclose their partners' HIV status leading to missing values. ^∗^*P* < 0.05 and ^∗∗^*P* < 0.01.

**Table 4:** Association between variables and willingness to use PrEP (*N* = 550).

Variables	Response categories	Crude OR (95% CI)		
		Willingness to use PrEP	Willingness to use oral PrEP	Willingness to use LAI-PrEP
Age (years)	18-24	1.0	1.0	1.0
	25-30	0.92 (0.60, 1.40)	0.90 (0.59, 1.36)	0.76 (0.50, 1.14)
	>30	0.86 (0.56, 1.31)	0.75 (0.50, 1.14)	0.74 (0.49, 1.13)
Education	Middle school or below	1.0	1.0	1.0
	University or above	1.36 (0.94, 1.97)	1.36 (0.95, 1.96)	1.01 (0.70, 1.46)
Monthly income (CNY)	<2,000	1.0	1.0	1.0
	2,000-4,999	1.10 (0.60, 2.02)	1.15 (0.63, 2.10)	0.77 (0.42, 1.42)
	5,000-9,999	1.29 (0.72, 2.33)	1.42 (0.80, 2.52)	0.86 (0.48, 1.54)
	>9,999	1.30 (0.67, 2.56)	1.28 (0.66, 2.46)	1.04 (0.54, 2.01)
History of STDs	No	1.0	1.0	1.0
	Yes	0.97 (0.52, 1.83)	0.85 (0.46, 1.58)	1.38 (0.75, 2.53)
Alcohol consumption	No	1.0	1.0	1.0
	Yes	1.21 (0.83, 1.78)	1.17 (0.80, 1.70)	1.47 (0.99, 2.16)^†^
Drug use	No	1.0	1.0	1.0
	Yes	1.31 (0.87, 1.98)	1.36 (0.90, 2.03)	0.95 (0.64, 1.41)
Self-reported HIV status of current sex partners^a^	Uncertain	1.0	1.0	1.0
	HIV negative	0.98 (0.63, 1.54)	0.96 (0.62, 1.49)	0.79 (0.51, 1.23)
	HIV positive	1.03 (0.41, 2.60)	0.65 (0.27, 1.56)	1.38 (0.57, 3.32)
Number of regular sexual partners in the past 6 months	0	1.0	1.0	1.0
	1-2	1.04 (0.71, 1.52)	1.10 (0.76, 1.60)	0.89 (0.61, 1.30)
	≥3	1.16 (0.68, 1.99)	1.18 (0.70, 1.99)	1.11 (0.67, 1.85)
Number of casual sexual partners in the past 6 months	0	1.0	1.0	1.0
	1-2	1.15 (0.62, 2.13)	1.07 (0.58, 1.98)	0.71 (0.39, 1.30)
	≥3	1.42 (0.76, 2.64)	1.28 (0.69, 2.37)	0.78 (0.42, 1.43)
Condom used during last anal sex	No	1.0	1.0	1.0
	Yes	1.17 (0.79, 1.75)	1.07 (0.72, 1.59)	1.19 (0.80, 1.78)
Sex with women	No	1.0	1.0	1.0
	Yes	0.97 (0.67, 1.41)	1.00 (0.70, 1.44)	1.21 (0.84, 1.73)
Ever used gay dating app in the past 6 months	No	1.0	1.0	1.0
	Yes	1.50 (1.05, 2.16)^∗^	1.41 (0.99, 2.01)^†^	1.07 (0.75, 1.53)
HIV testing in the past 12 months	0	1.0	1.0	1.0
	1-2 times	1.12 (0.73, 1.73)	1.05 (0.68, 1.61)	1.01 (0.66, 1.55)
	3 times or more	1.43 (0.85, 2.40)	1.12 (0.68, 1.85)	1.35 (0.82, 2.22)
STD testing in the past 12 months	0	1.0	1.0	1.0
	1-2 times	1.19 (0.81, 1.75)	1.12 (0.77, 1.63)	1.33 (0.92, 1.93)
	3 times or more	1.45 (0.79, 2.66)	1.30 (0.73, 2.33)	1.20 (0.68, 2.11)
Circumcision	No	1.0	1.0	1.0
	Yes	1.03 (0.64, 1.65)	0.94 (0.59, 1.48)	1.21 (0.77, 1.91)
Ever participated in HIV- or STD-related studies	No	1.0	1.0	1.0
	Yes	1.96 (1.29, 2.97)^∗∗^	1.65 (1.11, 2.45)^∗^	1.51 (1.04, 2.19)^∗^
Heard of PrEP	No	1.0	1.0	1.0
	Yes	3.07 (2.09, 4.51)^∗∗^	2.84 (1.96, 4.11)^∗∗^	2.03 (1.43, 2.88)^∗∗^

CI: confidence interval; CNY: Chinese Yuan, CNY 2,000 equal to USD 302 and CNY 1,512 equal to USD 1,512; STDs: sexually transmitted diseases; CDC: Centers for Disease Control; PrEP: preexposure prophylaxis. ^a^The last sex partners who are in a regular or causal relationship with participants; 8 MSM refused to disclose their partners' HIV status leading to missing values. ^†^*P* < 0.1, ^∗^*P* < 0.05, and ^∗∗^*P* < 0.01.

**Table 5:** Factors associated with willingness to use PrEP among MSM in southern China (*N* = 550).

Variables	Response categories	Adjusted OR (95% CI)		
		Willingness to use PrEP	Willingness to use oral PrEP	Willingness to use LAI-PrEP^a^
Alcohol consumption	No	/	/	1.0
	Yes	/	/	1.30 (0.86, 1.96)
Ever used gay dating app in the past 6 months	No	1.0	1.0	/
	Yes	1.39 (1.02, 1.97)^∗^	1.27 (0.88, 1.84)	/
Ever participated in HIV- or STD-related studies	No	1.0	1.0	1.0
	Yes	1.90 (1.24, 2.92)^∗∗^	1.59 (1.06, 2.39)^∗^	1.51 (1.03, 2.21)^∗^
Heard of PrEP	No	1.0	1.0	1.0
	Yes	2.96 (2.06, 4.54)^∗∗^	2.75 (1.90, 4.00)^∗∗^	2.00 (1.41, 2.85)^∗∗^

CI: confidence interval; STDs: sexually transmitted diseases; PrEP: preexposure prophylaxis. ^a^Adjusted for alcohol consumption. ^∗^*P* < 0.05 and ^∗∗^*P* < 0.01.

## Data Availability

The data used to support the findings of this study are available from the corresponding author upon request.
